# Letter to the Editor of IOVS From Joseph L. Demer and Robert A. Clark Regarding Joel M. Miller, “EOM Pulleys and Sequelae: A Critical Review”

**DOI:** 10.1167/iovs.61.6.10

**Published:** 2020-06-05

**Authors:** Joseph L. Demer, Robert A. Clark

**Affiliations:** 1 University of California -Los Angeles, Los Angeles, California, United States; 2 Stein Eye Institute, University of California -Los Angeles, Los Angeles, California, United States. E-mail: jld@jsei.ucla.edu.

**Keywords:** Listing's Law, orbital pulley, magnetic resonance imaging, compartmentalization

This letter responds to the recent review by Joel M. Miller,^1^ purportedly refuting the active pulley hypothesis (APH) of ocular kinematics, and criticizing with panoramic scope the methods and findings of publications from our research group.

Dr. Miller himself was prescient in his 2007 review, entitled “Understanding and Misunderstanding Extraocular Muscle Pulleys,” when he wrote: “We will find that most critiques of pulley theory are incorrect, being based on gross misunderstanding or directed at abandoned hypotheses.”^2^ That statement is unfortunately characteristic of Dr. Miller's own 2019 review.^1^

The core of the APH, as Demer first articulated it in 2000^3^ and elaborated in the Friedenwald Lecture paper in 2003^4^ is that the orbital layer (OL) of each rectus extraocular muscle (EOM) inserts on that EOM's pulley and shifts the pulley posteriorly during EOM contraction. This notion, which Miller later termed the concept of “coordinated pulley control,” does not require any relative shift at all between each EOM's OL and oculorotary global layer (GL), as Miller clearly stated in his 2007 review when he wrote:

There is nothing in the notion of coordinated active pulleys about independent control or differential motion of orbital and global lamina. Laminar distinctions are merely references to known anatomy. Nothing about coordinated APH kinematics would change if all fibers were coupled to both the pulley sleeve and the sclera.^2^

It is thus more than puzzling that Miller's 2019 review absolutely contradicts his foregoing statement with the assertion that “The absence of neural support for independent control (of OL and GL) clearly disconfirms the APH.”^1^ Although Demer's initial publication of the APH also offered up the possibility of differential pulley position control of to implement the non-Listing's kinematics of the vestibulo-ocular reflex, Demer quickly abandoned that view, as Miller acknowledged in his 2007 review.^2^ In this context, Miller is remarkably erroneous in his assertion in the 2019 review that “…the APH implausibly requires relative movements of OL and GL sufficiently large to translate pulleys and alter the actions of muscles passing through them.” It seems that Miller's review has rather baldly misrepresented the APH, as Demer proposed it and Miller correctly explained it in 2007,^2^ to make the astonishing claim that the APH has now been disconfirmed.

Even if mechanical independence among all EOM fibers were required to sustain the APH (And just to be absolutely clear, laminar independence is not required at all to implement Listing's law.), what is the quality of the evidence cited in Miller's 2019 review to exclude any independence? Miller's review casually dismissed extensive functional studies from the Demer laboratory showing minimal lateral force transmission among arbitrary groups of bovine EOM muscle^5^ and tendon^6^ fibers during external loading, and EOMs actively contracting ex vivo.^7^ The basis for this dismissal of the only existing functional evidence on the question is speculation about possible effects of removing epimysium connective tissues external to EOMs prior to mechanical testing,^8^ and a letter to a journal editor alleging use of excessive tensile forces during testing.^9^ The epimysium is synonymous with the muscle capsule, which in cow is a small fraction of 1 mm thin, relative to the thick 5- to 10-mm transverse dimensions of a bovine EOM. It is simply implausible to propose that the necessary, careful dissection of the thin external muscle capsule fundamentally disturbed internal connective tissue coupling among all of the many thousands of fibers deep within the EOM, or that removal of the thin external capsule somehow surrounding both layers destroyed Miller's putative, tight mechanical connection between GL and OL. For tensile testing, the Demer laboratory uses force sensors on frictionless air bearings capable of resolving 0.02 mN (2-mg force) tension applied to EOM or tendon.^7^ Because all specimen force loading was monitored as it gradually increased from zero, there was no chance of missing coupling effects that might exist only at low force.

Miller's 2019 review asserts that differential contractile forces can develop across an EOM's width, yet somehow substantial differential movements cannot occur. The meaningfulness of such distinction depends on the definition of “substantial.” The concept of muscle “contraction” necessarily implies some shortening, somewhere and on some scale. Different muscle fibers that contract differently, as due to different recruitment threshold, or fiber type, or laminar insertion, must shorten differently during activation, and therefore could not in principle be monolithically united to all other fibers while exerting, as Miller implies, significant differential forces. The magnitude of differential movement need not be very large to be physiologically important. For example, a 1-mm differential movement in a typical rectus EOM 40 mm long would only represent a 2.5% differential, yet might plausibly mediate a physiologically significant vergence eye rotation. The Demer laboratory has published a series of papers demonstrating by magnetic resonance imaging (MRI) the existence of differential compartmental function in EOMs under a wide variety of conditions, and carefully compared a range of MRI metrics indicating EOM contractility, including both posterior partial volume (PPV) and maximum cross sectional area. The Demer laboratory published a systemic comparison indicating that all measures reflect contractility and typically concord, but that PPV is most robust.^10^ Our analysis was not subject to the confound alleged in Figures 1 and 2 of Miller's review; we consistently assigned image plane numbers relative to the location of the globe-optic nerve junction (G-ONJ) in central gaze. Moreover, many of our MRI studies, for example involving ocular counterrolling^11^^,^^12^ and various forms of vergence,^13^^–^^16^ involve zero or sufficiently small horizontal or vertical eye rotations that cause no material change in anteroposterior position of the G-ONJ at all, and thus could not have been confounded in the way Miller so broadly alleges. No scientific technique is free of limitations and artifacts, but the quantitative measures we have used in our more than 105 peer-reviewed research publications applying MRI to the ocular motor system have applied adequate methodology to support the APH and the compartmentalization hypothesis. We have performed much self-replication in the process, supporting the robustness of the findings. Space does not permit specific refutation of every individual criticism offered by Miller's wide-ranging review, but we are confident that none of them undermines our conclusions as published.

Miller extended his review to allege lack of rigor, broadly suggesting various implied biases and statistical inadequacies, and lack of confirmation of “studies from the Demer lab.”^17^ All of our experimental studies did undergo critical peer review, mainly by journals such as *IOVS* and *Journal of Neurophysiology*, which was not the case for the “personal communication” and letters to the editor upon which Miller relies to argue for refutation of the APH. The lack of “confirmation” of our work should not be understood, as might be erroneously implied from Miller's review, to be disconfirmation; rather, no other laboratory has attempted these demanding experiments, so no one should assume as Miller implies that the experiments have been improperly performed or interpreted. In his conclusion, Miller seems to demand a standard of experimental design appropriate to a clinical trial of a drug or device, for example including only testing explicit a prior hypothesis and analysis by multiple observers. Miller's own work typically, and that of the studies upon which his new review relies, have not conformed to the stringent standard he advocates for multiple analysts^18^^,^^19^ or robust statistical adequacy in a large number of subjects.^18^^,^^20^ But to be fair to the field, ocular motor physiology is exploratory basic science and not a clinical trial, such that many experiments, such as those in nonhuman primates, are so costly and time-consuming that very large sample sizes and rigid prospective protocols are seldom possible. And the reader should also keep in mind that the alternative to the current, imperfect body of scientific data is continued conceptualization of the ocular motor system through intuition, assumptions, and untested dogma.

Miller's review concludes with suggestions for future directions for study of EOM compartmentalization. We agree that it would be valuable to perform anatomical and functional studies of possible segregated motor neuron pools in the brainstem for control of the OL and GL, and for transverse EOM compartments. Our preliminary work in this area, although suggestive, is as yet insufficient for publication. We would welcome the entry of primate ocular motor laboratories into this important area of investigation, and hope that Miller will join in this effort. We have recently adopted increasing levels of automation for analysis of MRI studies of EOM function, and are investigating artificial intelligence for image segmentation. However, absence of such methods does not disconfirm the APH, which remains not only tenable, but the only explanation for fundamental ocular motor physiology such as Listing's law.^4^

## References

[1] Miller JM EOM pulleys and sequelae: a critical review. *Inv Ophthalmol Vis Sci*. 2019; 60: 5052–5058. 10.1167/iovs.19-28156 31800963

[2] Miller JM Understanding and misunderstanding extraocular muscle pulleys. *J Vision*. 2007; 7: 1–15. 10.1167/7.11.10 17997665

[3] Demer JL, Oh SY, Poukens V Evidence for active control of rectus extraocular muscle pulleys. *Invest Ophthalmol Vis Sci*. 2000; 41: 1280–1290. 10798641

[4] Demer JL. Pivotal role of orbital connective tissues in binocular alignment and strabismus. The Friedenwald lecture. *Invest Ophthalmol Vis Sci*. 2004; 45: 729–738. 14985282 10.1167/iovs.03-0464

[5] Shin A, Yoo L, Chaudhuri Z, Demer JL Independent passive mechanical behavior of bovine extraocular muscle compartments. *Inv Ophtalmol Vis Sci*. 2012; 53: 8414–8423. 10.1167/iovs.12-10318 PMC4113332 23188730

[6] Shin A, Yoo L, Demer JL Biomechanics of superior oblique Z-tenotomy. *J AAPOS*. 2013; 17: 612–617. 24321425 10.1016/j.jaapos.2013.09.004 PMC3858822

[7] Shin A, Yoo L, Demer JL Independent active contraction of extraocular muscle compartments. *Inv Ophtalmol Vis Sci*. 2015; 56: 199–206. 10.1167/iovs.14-15968 PMC4290554 25503460

[8] McLoon LK, Vicente A, Fitzpatrick KR, et al. Composition, architecture, and functional Implications of the connective tissue network of the extraocular muscles. *Invest Ophthalmol Vis Sci*. 2018; 59: 322–329. 29346490 10.1167/iovs.17-23003 PMC5773232

[9] Holt DG, Morrison DG, Donahue SP Biomechanics of superior oblique Z-tenotomy. *J AAPOS*. 2014; 18: 408. 10.1016/j.jaapos.2014.03.002 25173909

[10] Clark RA, Demer JL. Functional morphometry of horizontal rectus extraocular muscles during horizontal ocular duction. *Invest Ophthalmol Vis Sci*. 2012; 53: 7375–7379. 22997285 10.1167/iovs.12-9730 PMC3481603

[11] Demer JL, Clark RA. Magnetic resonance imaging of human extraocular muscles during static ocular counter-rolling. *J Neurophysiol*. 2005; 94: 3292–3302. 16033934 10.1152/jn.01157.2004

[12] Clark RA, Demer JL Differential lateral rectus compartmental contraction during ocular counter-rolling. *Inv Ophthalmol Vis Sci*. 2012; 53: 2887–2896. 10.1167/iovs.11-7929 PMC3367472 22427572

[13] Demer JL, Clark RA. Functional anatomy of extraocular muscles during human vergence compensation of horizontal heterophoria. *J Neurophysiol*. 2019; 122: 105–117. 31042451 10.1152/jn.00152.2019 PMC6689778

[14] Demer JL, Clark RA. Functional anatomy of human extraocular muscles during fusional divergence. *J Neurophysiol*. 2018; 120: 2571–2582. 30230991 10.1152/jn.00485.2018 PMC6295544

[15] Demer JL, Clark RA. Magnetic resonance imaging demonstrates compartmental muscle mechanisms of human vertical fusional vergence. *J Neurophysiol*. 2015; 113: 2150–2163. 25589593 10.1152/jn.00871.2014 PMC4416603

[16] Demer JL, Clark RA, Crane BT, et al. Functional anatomy of the extraocular muscles during vergence. *Prog Brain Res*. 2008; 171: 21–28. 18718278 10.1016/S0079-6123(08)00604-3 PMC2881303

[17] Miller JM. Invest Ophthallmol Vis Sci. *EOM Pulleys and Sequellae: A Critical Review*. 2019; 60: 5052–5058. 10.1167/iovs.19-28156 31800963

[18] Miller JM Functional anatomy of normal human rectus muscles. *Vision Res*. 1989; 29: 223–240. 2800349 10.1016/0042-6989(89)90126-0

[19] Demer JL, Miller JM. Magnetic resonance imaging of the functional anatomy of the superior oblique muscle. *Invest Ophthalmol Vis Sci*. 1995; 36: 906–913. 7706039

[20] Miller JM, Davison RC, Gamlin PD Motor nucleus activity fails to predict extraocular muscle forces in ocular convergence. *J Neurophysiol*. 2011; 105: 2863–2873. 21451064 10.1152/jn.00935.2010 PMC3118740

